# A Complex Relationship between TRAF3 and Non-Canonical NF-κB2 Activation in B Lymphocytes

**DOI:** 10.3389/fimmu.2013.00477

**Published:** 2013-12-20

**Authors:** Wai W. Lin, Joanne M. Hildebrand, Gail A. Bishop

**Affiliations:** ^1^Graduate Program in Immunology, University of Iowa, Iowa City, IA, USA; ^2^Department of Microbiology, University of Iowa, Iowa City, IA, USA; ^3^Department of Internal Medicine, University of Iowa, Iowa City, IA, USA; ^4^VA Medical Center, Iowa City, IA, USA

**Keywords:** TRAF3, NF-κB, B cell, CD40, BAFF

## Abstract

The adaptor protein TRAF3 restrains B cell activating factor receptor (BAFFR) and CD40-mediated activation of the NF-κB2 pathway in B cells. Mice lacking TRAF3 specifically in B cells revealed the critical role of TRAF3 in restraining homeostatic B cell survival. Furthermore, loss-of-function mutations of the *traf3* gene have been associated with human B cell malignancies, especially multiple myeloma (MM). It has been proposed that receptor-induced TRAF3 degradation leads to stabilization of the NF-κB inducing kinase (NIK), and subsequent NF-κB2 activation. However, it is unclear how receptor-mediated TRAF3 degradation or loss-of-function contributes to B cell-specific NF-κB2 activation. In the current study, we employed two complementary models to address this question. One utilized a mutant *traf3* gene found in a human MM-derived cell line called LP1. The LP1 mutant TRAF3 protein lacks the TRAF-N and TRAF-C domains. Consistent with the paradigm described, expression of LP1 TRAF3 in B cells promoted higher basal levels of NF-κB2 activation compared to Wt TRAF3. However, LP1 did not associate with TRAF2, CD40, or BAFFR, and no LP1 degradation was observed following receptor engagement. Interestingly, LP1 showed enhanced NIK association. Thus, TRAF3 degradation becomes dispensable to activate NF-κB2 when it is unable to associate with TRAF2. In a second model, we examined several mutant forms of BAFFR that are unable to induce NF-κB2 activation in B cells. Signaling to B cells by each of these BAFFR mutants, however, induced levels of TRAF3 degradation similar to those induced by Wt BAFFR. Thus, in B cells, receptor-mediated TRAF3 degradation is not sufficient to promote NF-κB2 activation. We thus conclude that there is not a simple linear relationship in B lymphocytes between relative levels of cellular TRAF3, induced TRAF3 degradation, NIK activation, and NF-κB2 activation.

## Introduction

Tumor necrosis factor receptor (TNFR) associated factor 3 (TRAF3) is an important adaptor molecule shown to regulate NF-κB2 activation induced by the TNFR superfamily molecules CD40 and B cell activating factor receptor (BAFFR), as well as other immune receptors (1–3). In B lymphocytes, CD40 and BAFFR-mediated signaling is important for regulation of maturation, survival, Ig class switch recombination, and antibody production (4, 5). Engagement of BAFFR with BAFF or interaction between CD40 and its ligand CD154 triggers the non-canonical NF-κB or NF-κB2 pathway [reviewed in Ref. (6)]. TRAF3 negatively regulates NF-κB2 activation (7, 8), and mouse B cells deficient in TRAF3 have substantially enhanced survival, which correlates with constitutive activation of the NF-κB2 pathway (9, 10). Understanding how TRAF3 regulates both NF-κB2 activation and homeostatic B cell survival is thus of great importance.

The NF-κB2 pathway is tightly regulated at steady state, and dysregulation of the pathway is frequently observed in hematological malignancies (11, 12). Gain or loss-of-function mutations of genes encoding key signaling molecules implicated in the NF-κB2 pathway have been observed in such tumors. In particular, mutations in NF-κB inducing kinase (NIK), TRAF3, TRAF2, and cellular inhibitors of apoptosis 1 and 2 (cIAP1, cIAP2) are highly associated with human multiple myeloma (MM) and MM-derived cell lines. Loss-of-function mutations of the *traf3* gene have been identified in 9–17% of MM patient cohorts (13, 14). Such mutations have also been identified in different subtypes of B cell lymphoma and Waldenström’s macroglobulinemia (15–17). Consistent with this, a portion of older mice deficient in TRAF3 specifically in B cells develop B cell lymphomas (18). Taken together, these studies provide strong evidence that loss or reduced expression of TRAF3 contributes to B cell malignancies.

A widely held paradigm suggests that the physical association between TRAF3, TRAF2, and NIK allows the TRAF2-cIAP E3 ubiquitin ligase complex to polyubiquitinate NIK (19, 20). This promotes proteasome-mediated degradation of NIK, and by so doing, restrains NF-κB2 activation (21). Engagement of CD40 or BAFFR on B cells is known to recruit TRAFs 2 and 3 to the plasma membrane lipid-raft compartment (22, 23). This recruitment initiates TRAF2-mediated polyubiquitination of both TRAFs 2 and 3, and their subsequent proteasome-mediated degradation (22, 24, 25). The paradigm mentioned above posits that this receptor-mediated TRAF3 degradation allows NIK stabilization, and thus NF-κB2 activation (26, 27). However, it remains unclear whether TRAF3 degradation is necessary and sufficient for NF-κB2 activation, in response to CD40 and BAFFR signaling, in B lymphocytes. The present study was designed to address this important mechanistic question. Utilizing two complementary approaches, we found that association between TRAFs 2 and 3 is important for CD40 and BAFFR-mediated TRAF3 degradation. However, degradation of endogenous TRAF3 was neither sufficient nor always required to induce CD40 or BAFFR-mediated activation of NF-κB2 in B cells.

## Materials and Methods

### Mice

A/J, BAFFR^−/−^, and Bcl2 transgenic (tg) mice with A/J congenic backgrounds were kindly provided by Dr. Colleen Hayes (University of Wisconsin, Madison, WI, USA). A/WySnJ mice, also on the A/J genetic background, were purchased from Jackson Laboratory (Bar Harbor, ME, USA). A/J, A/WySnJ, and BAFFR^−/−^ mice were bred with Bcl2 tg mice to circumvent mature B cell developmental defects in A/WySnJ and BAFFR^−/−^ mice (28, 29). All mice had one allele of the *Bcl2* transgene, and were used at 10–12 weeks of age as a source of B cells for experiments. Mice were maintained under pathogen-free conditions at the University of Iowa. Use of mice in this study was according to a protocol approved by The University of Iowa Animal Care and Use Committee.

### Cell lines

The mouse B cell lines M12.4.1 (30), CH12.LX, and its TRAF3-deficient subclones have been previously described (31, 32). B cell lines stably transfected with hybrid human CD40-mouse BAFFR constructs described below were maintained in B cell medium containing RPMI 1640 (Life Technology, Grand Island, NY, USA) with 10 μM 2-βmercaptoethanol (Sigma Aldrich, St. Louis, MO, USA), 10% heat-inactivated FCS (Atlanta Biologicals, Atlanta, GA, USA), 2 mM l-Glutamine (Life Technologies), 100 U/ml of Penicillin Streptomycin antibiotics (Life Technologies) (BCM10). Medium additionally contained 400 μg/ml of G418 disulfate (Research Products International, Mount Prospect, IL, USA) for subclones expressing transfected hCD40-BAFFR constructs, and both G418 and 200 μg/ml of hygromycin (Life Technologies) for subclones expressing FLAG-tagged TRAF3 or the LP1 mutant TRAF3.

### DNA constructs and transfections

Plasmids encoding a mutant BAFFR from the A/WySnJ mouse and a mouse BAFFR lacking the C-terminal eight amino acids (ΔC) were kindly provided by Drs. Colleen Hayes and Christopher Mayne. These plasmids were used as a source of BAFFR to produce cDNA encoding a chimeric molecule consisting of the extracellular portion of human CD40 and the transmembrane and intracellular portions of mouse Wt BAFFR, the BAFFR mutant of the A/WySnJ mouse, or mouse BAFFR lacking the C-terminal 8 amino acids (ΔC). These chimeric constructs were subcloned into the mammalian expression vector pRSV5.neo for stable expression in CH12.LX or M12.4.1 cells (33). cDNAs encoding FLAG-tagged human Wt TRAF3, or the truncated TRAF3 mutant identified in the human MM cell line LP1 (14) were subcloned into a variant of the pRSV5.neo plasmid with a sequence containing the binding element of the repressor of the bacterial Lac operon in the promoter, as previously described (33). This allows Isopropyl β-d-1-thiogalactopyranoside (IPTG)-inducible, stable TRAF3 expression in a CH12.TRAF3^−/−^ cell line that stably expresses the Lac repressor (32, 33). There is thus no endogenous TRAF3 in cells expressing Wt or LP1 TRAF3 in these studies.

### Antibodies and reagents

Isopropyl β-d-1-thiogalactopyranoside was purchased from Sigma Chemical Co. (St. Louis, MO, USA). Rabbit anti-phospho-jun kinase (JNK) Ab, rabbit anti-phospho-p38 Ab, rabbit anti-pIκBα Ab, rabbit anti-total IκBα Ab, rabbit anti-NIK Ab, and rabbit anti-p100/p52 Ab were purchased from Cell Signaling Technology (Danvers, MA, USA). Rabbit anti-TRAF2 Ab was purchased from Medical and Biological Laboratories (Woburn, MA, USA). Anti-FLAG M2 Ab for immunoprecipitation, anti-FLAG M2-HRP Ab for immunoblotting, and mouse anti β-actin Ab were purchased from Sigma. Rabbit anti-JNK Ab, mouse anti-YY1 (H-10) Ab, and rabbit anti-mouse CD40 (M-20) were purchased from Santa Cruz Biotechnology (Dallas, TX, USA). Production of Hi Five (HiV) strain insect cells infected with Wt baculovirus or baculovirus encoding human CD154 or mouse CD154 is described in (34). Rat anti-mouse CD40 mAbs (1C10 and 4F11) were produced in our laboratory from their respective hybridomas as described (32), kindly provided by Dr. Frances Lund (University of Alabama, Birmingham, AL, USA). The isotype control rat total IgG Ab was purchased from Southern Biotech (Birmingham, AL, USA) and mouse IgG1 control Ab from eBioscience (San Diego, CA, USA). Goat anti-BAFFR Ab for immunoprecipitation was purchased from R&D Systems (Minneapolis, MN, USA), and rabbit anti-BAFFR Ab for immunoblotting was purchased from Abcam (Cambridge, MA, USA). HRP-conjugated goat anti-mouse IgG and goat anti-rabbit IgG secondary Abs were purchased from Jackson ImmunoResearch Laboratories (West Grove, PA, USA).

### Cell isolation and assays to measure receptor signaling and TRAF3 degradation

Resting splenic B cells were obtained as previously described (9). Briefly, high density splenic B cells were isolated by centrifugation through a Percoll density gradient (GE Life Sciences, Pittsburg, PA, USA) followed by anti-CD43 Ab-mediated negative selection, using a magnetic bead kit (Miltenyi Biotec, Auburn, CA, USA), according to the manufacturer’s protocol. 5 × 10^6^ splenic B cells or 2 × 10^6^ CH12.LX or M12.4.1 cells were stimulated as indicated in the Figure legends. After stimulation, cells were lysed and cytosolic and nuclear extracts were prepared as described in Xie et al. (35) to detect NF-κB2 activation via nuclear translocation of p52. For proximal signaling assays and TRAF3 degradation assays, 1–2 × 10^6^ cells were stimulated for indicated times at 37°C. After stimulation, cells were lysed in 100 μl 2× SDS-PAGE loading buffer (1% SDS, 2% β-mercaptoethanol, 62.5 mM Tris, pH 6.8). Lysates were sonicated using a Branson Sonifier 250 (VWR International, Radnor, PA, USA) with 10 pulses at 90% duty cycle, output 1.5. Samples were boiled for 10 min at 95°C prior to gel loading.

### Immunoprecipitation

CH12.TRAF3^−/−^ cells expressing LacR and stably transfected with inducible FLAG-tagged Wt or LP1 mutant TRAF3 were cultured with BCM10 containing 100 μM IPTG overnight to induce TRAF3 expression. 2 × 10^7^ cells were resuspended in 1 ml BCM10 and stimulated with 10 μg/ml of hamster anti-mouse CD40 mAb (HM40.3, eBioscience) or 500 ng/ml of recombinant BAFF (Peprotech, Rocky Hill, NJ, USA) for the indicated times. Cells were pelleted and lysed in lysis buffer (0.5% Triton X, 40 mM Tris, 100 mM NaCl, 1 mM MgCl_2_, 1 mM CaCl_2_) containing complete protease inhibitor cocktail (Roche, Germany), 0.05 mg of DNAse I (Roche), and 2 mM Na_3_VO_4_. Whole cell lyses were incubated with mouse anti-FLAG Ab (Sigma) or control IgG1 Ab overnight at 4°C with constant agitation. The immune complex was precipitated with Dyna Protein G beads (Life Technologies), washed and resuspended in SDS-PAGE loading buffer, and heated to 95°C for 10 min.

### Immunoprecipitation from detergent-insoluble plasma membrane fractions

3 × 10^7^ CH12.LX cells were stimulated at 37°C with 500 ng/ml recombinant BAFF (Peprotech) or 3 × 10^6^ HiV insect cells infected with Wt baculovirus, or baculovirus expressing mouse CD154 (34). These insect cells, which grow at 25°C, die and serve as a source of CD154-expressing membranes at 37°C. Cells were lysed using buffer containing 1% Brij58 (Thermo Scientific, Rockford, IL, USA), 150 mM NaCl, 20 mM Tris, 50 mM β-glycerophosphate, 1 mM MgCl_2_, 1 mM CaCl_2_, protease inhibitor cocktail, and 2 mM Na_3_VO_4_. The Brij58-insoluble cell fractions were separated by centrifugation at 14,000 × *g* for 30 min at 4°C and solubilized using 1% Triton X-100 and 0.1% SDS-containing buffer. This solubilized cholesterol-rich membrane fraction (22) was subjected to immunoprecipitation using rat anti-mouse CD40 mAbs (an equal mixture of the clones 1C10 and 4F11) (36), goat polyclonal anti-BAFFR Ab, or appropriate isotype control antibodies. To immunoprecipitate the receptor signaling complex, Protein G Dyna beads, pre-incubated with goat anti-rat Ab (Jackson) for 30 min at room temperature, or uncoupled beads were incubated with solubilized membrane fractions for 2 h at 4°C with constant agitation. After washing, the immunoprecipitate was deglycosylated using PNGaseF (NEB) and boiled in SDS-PAGE loading buffer.

### Immunoprecipitation of hCD40-BAFFR chimeric molecules

3 × 10^7^ cells CH12.LX cells that stably express hCD40-BAFFR (Wt), hCD40-A/WySnJ (A/WySnJ), hCD40-ΔC, or hCD40-BAFFR chimeric molecules with a mutated TRAF3 binding site (AVAAA) were stimulated with Protein G Dyna Beads (Life Technologies) conjugated with agonistic mouse anti-hCD40 mAb or isotype control Ab for the indicated time points at 37°C. Abs were crosslinked to the beads using DSS crosslinker (Thermo Scientific) according to the manufacturer’s recommended protocol. Immunoprecipitation of hCD40-BAFFR chimeric molecules was performed as previously described (37).

### Western blots

Proteins were resolved on SDS-PAGE gels and transferred to PVDF membranes (Millipore, Billerica, MA, USA). Membranes were blocked in 5% milk in TBST (120 mM NaCl, 0.08% Tween 20, and 40 mM Tris) for 1 h at room temperature, and incubated overnight at 4°C in primary Ab. Blots were washed in TBST, incubated with secondary Abs for 2 h at room temperature and developed using Supersignal West Pico (Thermo Scientific). Western blot chemiluminescence was read with an LAS-4000 low-light camera and analyzed with Multi Gage software (Fujifilm Life Science, Edison, NJ, USA).

### Statistical analysis

*p*-Values were generated by Student’s *t*-test (unpaired, two-tailed, at 95% confidence interval).

## Results

### Receptor-induced degradation of Wt vs. LP1 TRAF3

**Figure 1:**
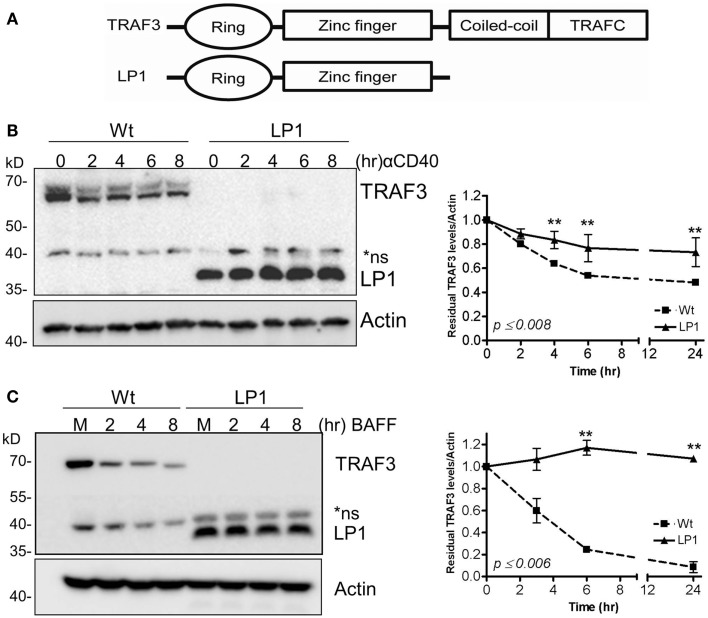
**Dissociation between CD40 and BAFF-mediated NF-κB2 activation and TRAF3 degradation**. **(A)** Schematic presentation of Wt and LP1 mutant TRAF3. **(B,C)** CH12.TRAF3^−/−^ cells stably transfected for inducible expression of FLAG-tagged Wt or LP1 mutant TRAF3 were treated overnight with IPTG to induce TRAF3 expression, as described in Section “Materials and Methods.” Induced cells were either untreated (M) or treated with isotype control Ab (C) or stimulated with 5 μg/ml of anti-CD40 mAb **(B)** or 200 ng/ml of recombinant BAFF **(C)** for the indicated times at 37°C. Whole cell lysates were resolved by SDS-PAGE and Western blots of gel probed with anti-FLAG Ab to assess relative cellular levels of Wt and LP1 TRAF3. Graphs depict mean values ± SEM of TRAF3 band intensities, normalized to actin, from immunoblots of three experiments. **Statistically significant differences between Wt and LP1 TRAF3, analyzed as in Section “Materials and Methods.” Numbers on the graph indicate the highest *p* values for individual time points.

We and others previously reported that B cell-specific loss of the *traf3* gene enhances the survival of B cells independent of cell proliferation (9, 10). We also demonstrated that this enhanced B cell-specific survival renders B cells independent of the soluble factor BAFF, and results in constitutive activation of the non-canonical NF-κB2 pathway (9). At the same time, several groups reported that loss-of-function mutations in the *traf3* gene are associated with human MM and enhanced NF-κB2 activity (13, 14). These studies identified various forms of TRAF3 mutations, from point mutations leading to single amino acid substitutions to large truncations. These mutations of *traf3* are highly correlated with frequent chromosomal translocations of chromosome 14, commonly seen in MM (13, 14). Among these is a TRAF3 mutation identified in the human MM LP1 cell line. The LP1 TRAF3 missense mutation results in production of a truncated TRAF3 lacking the TRAF-N and TRAF-C domains (Figure 1A), which we predict would fail to associate with TRAF2 (19) and thus not undergo receptor-mediated degradation.

To test our prediction that the LP1 TRAF3 mutant fails to undergo receptor-mediated degradation, we established CH12.TRAF3^−/−^ subclones with stable, IPTG-inducible expression of FLAG-tagged Wt or LP1 TRAF3. Cells were stimulated via CD40 or BAFFR, known strong activators of TRAF2-dependent TRAF3 degradation (22, 24, 25). Based on the results shown in Figures 1B,C, LP1 was only minimally degraded after stimulation with CD40 or BAFFR, compared to Wt TRAF3.

### Receptor-induced NF-κB2 and JNK activation in LP1-expressing cells

**Figure 2:**
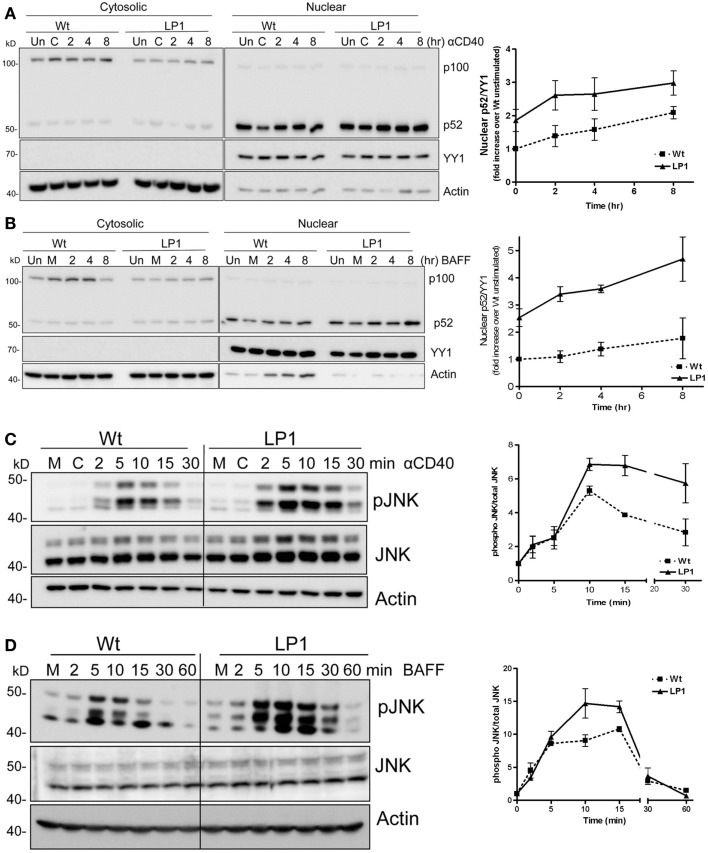
**Impact of TRAF3 on CD40 and BAFFR-mediated NF-κB2 and JNK activation**. CH12.TRAF3^−/−^ cells were induced to express Wt or mutant LP1 TRAF3 as in Figure 1. **(A,B)** Cells were untreated (M) or treated with isotype control Ab (C) or stimulated with 5 μg/ml of anti-mouse CD40 mAb (HM40.3) **(A)** or 200 ng/ml of recombinant BAFF **(B)** for the indicated times at 37°C. Uninduced cells were also included as control (Un). Cytosolic and nuclear extracts of cell lysates were prepared and immunoblotted for the NF-κB2 precursor protein p100 and its cleavage product p52, with probes for actin and YY1 serving as loading controls for cytoplasmic and nuclear protein levels respectively. YY1 levels were used to normalize nuclear p52 values shown quantitatively in graphs. Representative blots from cells stimulated via CD40 **(A)** or BAFFR **(B)** are shown. Graphs in **(A,B)** show mean values ± SEM of fold increases in normalized band intensities of nuclear p52 from five (CD40) or four (BAFF) independent experiments. Values from LP1-expressing cells were divided by values from Wt TRAF3-expressing unstimulated cells. **(C,D)** 1 × 10^6^ cells were stimulated with 10 μg/ml of anti-mouse CD40 mAb or 500 ng/ml of BAFF for the indicated times. Whole cell lysates were prepared, and Western blots probed for phosphorylated JNK (pJNK), total JNK (JNK), and actin. Representative blots are presented. The corresponding graphs represent mean values for band intensities of pJNK divided by total JNK ± SEM from two (BAFF) or three (CD40) independent experiments.

A widely held paradigm is that TRAF3 degradation induced by CD40 or BAFFR ligation allows NIK stabilization, which then activates the NF-κB2 pathway (20, 26, 27). This paradigm predicts that TRAF3^−/−^ cells expressing LP1 would fail to undergo receptor-induced NF-κB2 activation, as LP1 is not able to undergo receptor-induced degradation. To investigate whether LP1 is inducing NF-κB2 activation independent of receptor-induced degradation, we provided CD40 or BAFF stimuli to CH12.TRAF3^−/−^ B cells induced to express either Wt or LP1 TRAF3. Consistent with TRAF3’s proposed role in NF-κB2 activation, induction of Wt TRAF3 expression reduced nuclear p52 from its elevated levels in TRAF3^−/−^ B cells. Because the LP1 TRAF3 mutant is a loss-of-function mutation, we predicted that LP1 TRAF3-expressing B cells will have constitutive nuclear p52 and there will be no further activation of the NF-κB2 pathway upon stimulation. Interestingly, however, induction of LP1 expression in TRAF3^−/−^ B cells also reduced nuclear p52 levels (Figures 2A,B). Induction of LP1 expression reduced basal nuclear p52 levels in TRAF3^−/−^ B cells. However, p52 levels remained ~twofold higher in LP1 compared to Wt TRAF3-expressing cells (Figures 2A,B), which is consistent with previous observations in myeloma cells and fibroblasts that the presence of LP1 TRAF3 mutant led to higher p52 levels (8, 14). Surprisingly, unlike TRAF3-deficient B cells, which further activate NF-κB2 in response to CD40 but not BAFFR stimulation (9), LP1-expressing B cells responded to both CD40 and BAFF stimulation with further nuclear p52 increases (Figures 2A,B). These data suggested that the TRAF3 mutant LP1 partially restrains NF-κB2 activation, and upon stimulation activates the NF-κB2 pathway independent of receptor-mediated LP1 degradation. Collectively, published studies and results presented here lead us to propose that induction of NF-κB2 activation in B cells does not solely depend on the absence of TRAF3, whether TRAF3 reduction is caused by receptor-induced degradation, mutation, or gene-targeted deletion.

**Figure 3:**
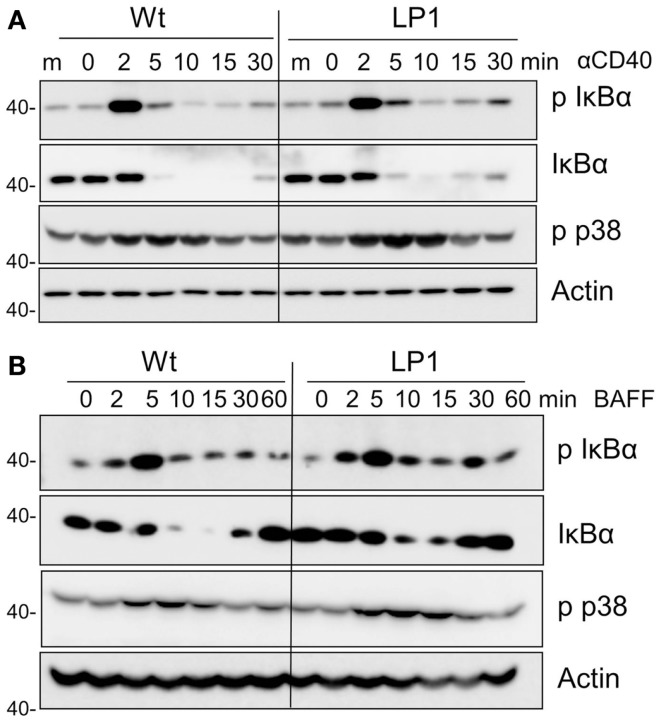
**Impact of TRAF3 on CD40 and BAFFR-mediated NF-κB1 and p38 MAP kinase activation**. CH12.TRAF3^−/−^ cells were induced to express Wt or mutant LP1 TRAF3 as in Figure 1. Cells were stimulated as described in Figures 2C,D via CD40 **(A)** or BAFFR **(B)**, and immunoblots of whole cell lysate samples were probed for phosphorylated IκBα (p IκBα), total IκBα, phospho p38 (p-p38), and actin (used as a control for p-p38 because antibodies to total p38 also recognize the phosphorylated form). Representative blots from three independent experiments are shown.

LP1 expression also resulted in enhanced c-JNK activation following BAFF stimulation (Figure 2D), and sustained and significantly enhanced JNK activation in response to CD40 signals (Figure 2C). In contrast, neither phosphorylation of the mitogen-activated kinase (MAPK) p38 nor activation of classical NF-κB1 was impacted by the LP1 mutation compared to Wt TRAF3 (Figure 3). These data indicate that the C-terminal domains of TRAF3 are important for restraining activation of the JNK pathway. Consistent with our prior report that complete loss of TRAF3 does not impact CD40-mediated NF-κB1 activation (9), LP1 expression did not result in enhanced NF-κB1 or p38 activation in response to CD40 or BAFFR signals. Canonical NF-κB1 activation was unaffected by LP1 TRAF3 expression, indicating that increased nuclear p52 levels in LP1-expressing cells were not due to NF-κB1-induced enhanced p100 expression.

### Association of TRAF3 with key signaling components in NF-κB2 activation

**Figure 4:**
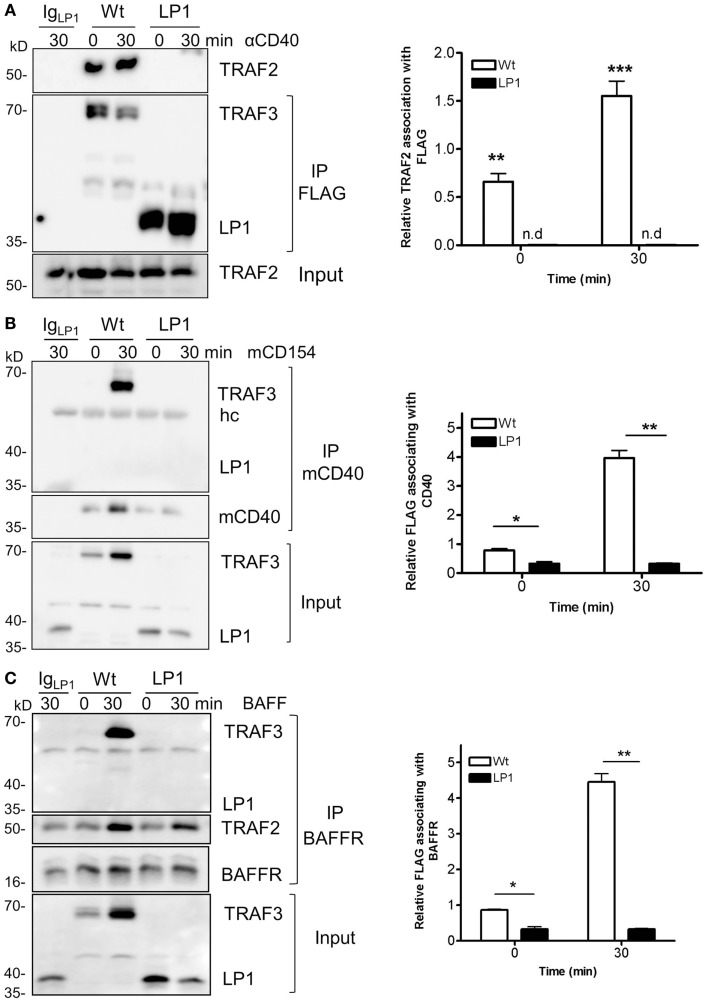
**Impaired association of LP1 TRAF3 with TRAF2, CD40, and BAFFR**. **(A)** CH12.TRAF3^−/−^ cells induced to express FLAG-tagged Wt or LP1 as in previous Figures were untreated or stimulated with 10 μg/ml of anti-CD40 mAb. Whole cell lysates were prepared as described in Section “Materials and Methods” and immunoprecipitated with anti-FLAG Ab and Western blots were probed for TRAF2 and FLAG. The bar graph presents quantification of relative amounts of TRAF3-associated TRAF2 from three independent experiments. **p = 0.001; ***p = 0.0005; n.d. = none detected. **(B)** Cells were stimulated with HiV insect cells infected with Wt baculovirus, or HiV cells expressing baculovirus-encoded mouse CD154. Detergent-insoluble plasma membrane fractions were prepared from cell lysates and subjected to immunoprecipitation with anti-mCD40 Ab as in Section “Materials and Methods.” Western blots were probed for FLAG and CD40. Graph represents quantification of mean values of relative amounts of TRAF3 associating with membrane CD40 from two different experiments. *p = 0.03; **p = 0.005. **(C)** Cells were untreated or cultured with 500 ng/ml of BAFF as indicated. Detergent-insoluble membrane fractions were isolated as above and anti-BAFFR Ab were used to immunoprecipitate the receptor signaling complex. Western blots of immunoprecipitates were probed for FLAG and BAFFR. The bar graph shows quantification of mean relative amounts of TRAF3 associated with BAFFR, from two different experiments. *p = 0.03; **p = 0.003.

TRAF3 is typically recruited to the cytoplasmic domains of CD40 and BAFFR, together with TRAFs, to mediate downstream signaling and biological functions (22, 23, 38). Recruitment to receptor cytoplasmic domains and association with TRAF2 and/or cIAP1/2 are thought to be required for K48-mediated ubiquitination, followed by proteasomal degradation of TRAF3 after receptor engagement (24, 25, 39, 40). To investigate the reason for the lack of receptor-mediated LP1 degradation, we assessed whether LP1 can associate with TRAF2. CH12.TRAF3^−/−^ B cells induced to express Wt or LP1 TRAF3 were stimulated through CD40, and Wt or LP1 TRAF3 was immunoprecipitated from cell lysates. Consistent with a previous report (19), the C-terminal TRAF-N and TRAF-C domains of TRAF3 were required for TRAF3 to associate with TRAF2, with or without CD40 stimulation (Figure 4A). Similar results were obtained with BAFF stimulation (data not shown).

To assess whether LP1 associated with either CD40 or BAFFR after stimulation, we isolated the lipid-raft enriched detergent-insoluble plasma membrane fractions of cells as described in Section “Materials and Methods,” and immunoprecipitated the receptor signaling complex. Figures 4B,C reveal that LP1 was not part of the CD40 or BAFFR signaling complexes, but Wt TRAF3 associated with the receptors upon stimulation. These data suggest that the observed lack of receptor-induced LP1 degradation reflects a requirement for recruitment of TRAF3 to the receptor cytoplasmic domains and association with TRAF2, to initiate the degradation process.

**Figure 5:**
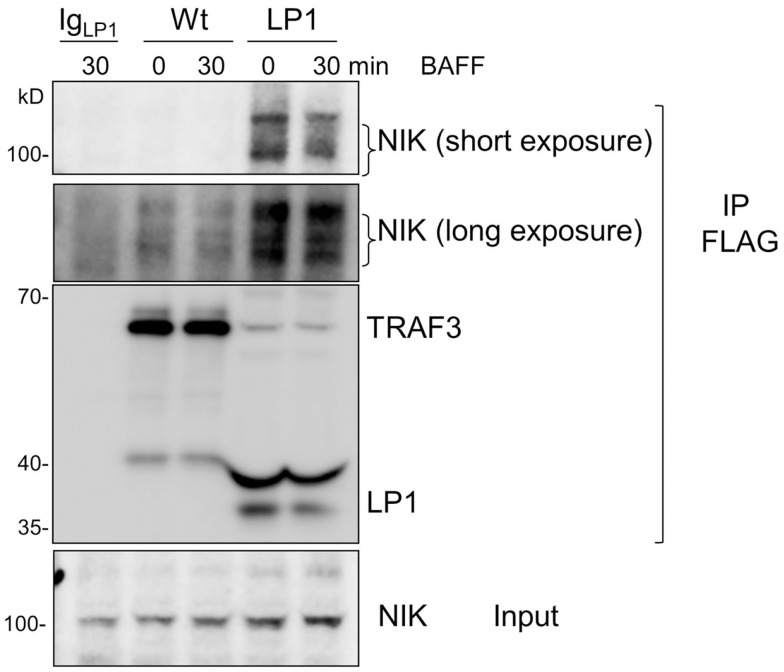
**Enhanced association of LP1 TRAF3 with NIK**. CH12.TRAF3^−/−^ cells expressing FLAG-tagged Wt or LP1 TRAF3 were untreated or stimulated with 500 ng/ml of BAFF. Whole cell lysates were prepared and immunoprecipitated with anti-FLAG Ab. Western blots of immunoprecipitates were probed for NIK and FLAG. Both short and long exposures of the blot are shown due to differences in NIK association between Wt and LP1 TRAF3. Data shown are representative of two similar experiments.

Strikingly, despite lack of receptor-mediated degradation of LP1 TRAF3, LP1-expressing B cells were capable of inducing NF-κB2 activation above the already high basal levels following receptor stimulation. To address this discrepancy with the prevailing paradigm, we investigated whether LP1 associates with NIK, a MAP kinase implicated as key to NF-κB2 activation (21). A previous report showed that TRAF3 physically interacts with NIK when both molecules are overexpressed in transformed epithelial cells, and this interaction requires the C-terminal residues from 424 to 543 of TRAF3 (20). The interaction between TRAF3 and NIK is proposed to allow the TRAF2 E3 ubiquitin ligase activity to constantly ubiquitinate NIK for proteosomal degradation (20, 26, 27). Hence, although LP1 was not degraded after receptor stimulation (Figure 1), if NIK fails to associate with LP1, this could stabilize NIK in LP1-expressing cells, ultimately leading to enhanced NF-κB2 activation. However, immunoprecipitated LP1 TRAF3 from unstimulated or BAFF-activated B cells displayed 10–16 fold *increased* association with NIK, compared to Wt TRAF3 (Figure 5). Notably, there was no drastic difference in total NIK accumulation in LP1 vs. Wt TRAF3-expressing B cells. It is important to note that the function of TRAF3 is highly context-dependent; TRAF3 plays very different roles with regards to distinct receptors and cell types [reviewed in Ref. (2)]. Thus, the discrepancy between the present findings and previous conclusions based upon experiments performed in epithelial cell lines (20) are likely due to these contextual differences. Collectively, the present results suggest that when tumor-derived mutant TRAF3 becomes resistant to receptor-mediated degradation, stabilization of cellular NIK levels is controlled by TRAF3 degradation-independent mechanisms to regulate NF-κB2 activation in B cells.

### Defective NF-κB2 activation via the mutant BAFFR of A/WySnJ B cells

**Figure 6:**
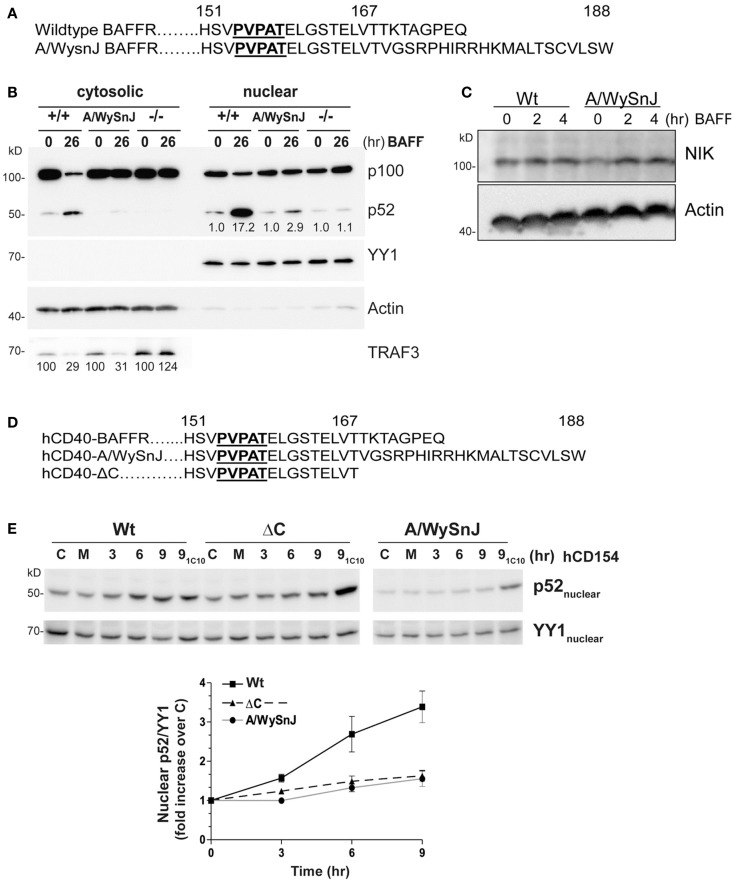
**Impaired NF-κB2 activation induced by mutant BAFFRs**. **(A)** Comparison of the C-terminal signaling domain amino acid sequences of Wt and A/WySnJ BAFFRs. Bold and underlined sequences indicate the TRAF3 binding motif, numbers above the sequences indicate the position of the amino acids. **(B)** Equal numbers of resting splenic B cells isolated from Wt (+/+), A/WySnJ, and BAFFR^−/−^ (−/−) mice were stimulated with 100 ng/ml of BAFF for 26 h. Cytosolic and nuclear fractions were prepared and subjected to SDS-PAGE as in Section “Materials and Methods.” Western blots of samples were probed for p100/p52, TRAF3, actin, and YY1, as in Figure 1. Numbers on the TRAF3 blot indicate mean values of the proportion of remaining TRAF3 relative to unstimulated controls of each genotype, from two independent experiments. Numbers on the blot of nuclear extracts represent averages of the fold increase over unstimulated control within each strain of mouse, from two independent experiments. **(C)** Equal numbers of splenic B cells isolated from Wt or A/WySnJ mice were stimulated with 200 ng/ml of BAFF for the indicated times. Whole cell lysates were immunoblotted for NIK and actin. Representative blots from two independent experiments are shown. **(D)** C-terminal amino acid sequences encoded by chimeric constructs of hCD40-BAFFR, used to stably transfect CH12.LX cells as described in Section “Materials and Methods.” **(E)** CH12.LX B cell subclones stably expressing various hCD40-BAFFR chimeras were treated with medium only (M), control HiV insect cells (C), or HiV insect cells expressing hCD154 for the indicated times. Anti-mCD40 agonistic mAb (1C10) treatment for 9 h served as an internal control for each subclone (9_1C10_). Nuclear extracts were prepared and Western blots probed for p100/p52, and YY1. Representative blots of three similar experiments are shown. The corresponding graph represents mean band intensity values of treated/control samples ± SEM of three independent experiments.

B cell activating factor receptor provides an important B cell survival signal, thought to require activation of the non-canonical NF-κB2 pathway (5). As a complementary experimental model to further examine the importance of receptor-mediated TRAF3 degradation for NF-κB2 activation in B cells, we isolated B cells from the A/WySnJ mouse. This mouse contains a spontaneous mutation in the *Baffr* gene called *Bcmd1* (B cell maturation defect-1) that leads to defects in survival and maturation of peripheral B cells (41). *Bcmd1* encodes a protein that shares ≥95% sequence homology with Wt BAFFR. Notably, the TRAF3 binding motif PVPAT remains intact in this mutant BAFFR (Figure 6A) (42). However, the last eight amino acids of Wt BAFFR are replaced by a 22-amino acid peptide sequence resulting from a proviral Intracisternal A-particle (IAP) retro-transposon insertion event (41). Although A/WySnJ mice have considerably reduced peripheral B cell numbers due to reduced B cell survival, the expression of tg Bcl2 restores the peripheral B cell compartment to near-normal levels (29). We thus utilized A/WySnJ-Bcl2 Tg mice for these experiments (designated A/WySnJ in Figures for simplicity). When we stimulated their splenic B cells with BAFF, the NF-κB2 pathway showed markedly defective activation compared to control mice of the parent strain (A/J) expressing tg Bcl2 (Figure 6B). Consistent with the concept that BAFF binding to BAFFR preferentially activates the NF-κB2 pathway (43), BAFFR^−/−^ B cells were not able to active NF-κB2 in response to BAFF stimulation (Figure 6B). In Figure 6B, we also noted that A/WySnJ BAFFR signaling induced TRAF3 degradation to a similar extent as did Wt BAFFR. We also examined the endogenous NIK protein levels in B cells of Wt and BAFFR mutant mice, and found that there were no differences in cellular NIK levels in A/WySnJ B cells as compared to Wt B cells (Figure 6C). These data suggest that TRAF3 degradation, leading to NIK stabilization, is not sufficient in itself to activate the non-canonical NF-κB2 pathway.

Because BAFF binds to three receptors expressed on B cells, BAFFR (44), BCMA (B cell maturation antigen) (45), and TACI (transmembrane activator and calcium modulator and cyclophilin ligand) (46), it is important to verify that the defective BAFF response of splenic B cells from the A/WysnJ mouse is due to its mutant BAFFR. We thus generated CH12.LX and M12.4.1 mouse B cell lines stably expressing chimeric receptors, containing the human (h)CD40 extracellular and transmembrane domains fused to the cytoplasmic domain of Wt BAFFR (Wt), the A/WySnJ mutant BAFFR, or BAFFR that lacks the last eight amino acids of the cytoplasmic domain (ΔC) (Figure 6D). The chimeric receptors allowed us to be certain that we were examining consequences of BAFFR signaling in these B cells. B cells were stimulated with HiV insect cells expressing hCD154, and NF-κB2 activation was analyzed. Stimulation with anti-mouse (m)CD40 agonistic Ab served as an internal control, showing that there was no global defect in CD40 signaling of cells expressing various chimeric molecules. Consistent with results from primary splenic B cells from A/WySnJ or control mice, B cells expressing hCD40-A/WySnJ BAFFR displayed defective NF-κB2 activation compared to hCD40-Wt BAFFR (Figure 6E). This result indicates that the signaling defect observed in splenic B cells is due to the dysfunctional BAFFR. Impaired activation of NF-κB2 was also observed with hCD40-ΔC BAFFR, indicating that the last eight amino acids of BAFFR are necessary for NF-κB2 activation, although they have been shown to be dispensable for B cell survival (29). This reveals the interesting information that pathways alternative to NF-κB2 can mediate BAFFR-induced B cell survival.

### TRAF3 degradation mediated by mutant BAFFRs

**Figure 7:**
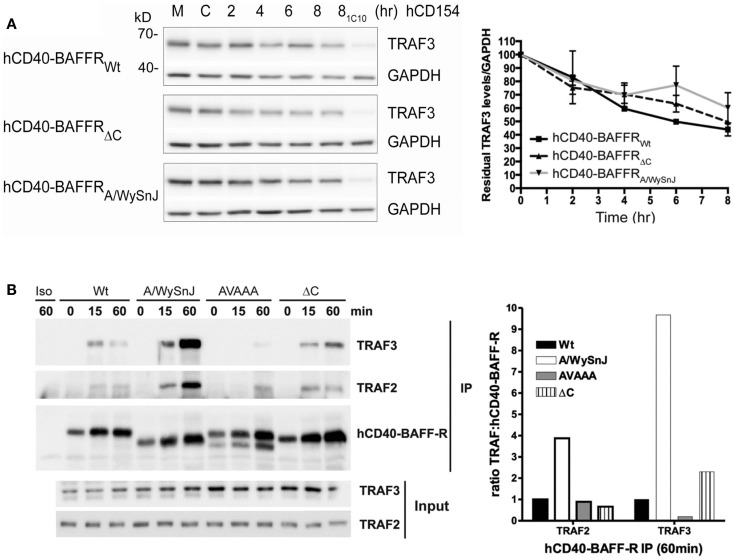
**Dissociation between relative TRAF3 degradation and NF-κB2 activation mediated by Wt vs. mutant BAFFRs**. **(A)** Subclones of the M12.4.1 B cell line stably transfected with various forms of hCD40-BAFFR chimeras shown in Figure 6C were untreated (M), or treated with HiV insect cells (C), or HiV insect cells expressing hCD154 for the indicated times, or anti-mCD40 agonistic Ab 1C10 for 8 h (8_1C10_). Whole cell lysates were prepared and subjected to SDS-PAGE as in Section “Materials and Methods.” Representative immunoblots of samples, probed for TRAF3 and GAPDH are presented. The graph depicts relative amounts of TRAF3, normalized to GAPDH. Values presented are mean ± SEM of data combined from three independent experiments. **(B)** CH12.LX B cells subclones as in Figure 6D and CH12.LX B cells that expressed hCD40-BAFFR with disrupted TRAF3 binding sites (AVAAA) were stimulated with anti-hCD40 mAb or isotype control (Iso) coated beads and immunoprecipitated as mentioned in Section “Materials and Methods.” The bead bound protein complexes were separated on SDS-PAGE and immunoblotted for TRAF2 and TRAF3. Representative blots from three independent experiments were shown. Bar graph represents relative levels of TRAF2 and TRAF3 normalized to hCD40-BAFFR chimera molecules.

To explore whether TRAF3 degradation status correlates with the impaired activation of NF-κB2 in cells expressing mutants of BAFFR, we stimulated B cell transfectants shown in Figure 6C through hCD40-BAFFR to induce BAFFR-mediated TRAF3 degradation (Figure 7A). TRAF3 was degraded similarly, regardless of which BAFFR cytoplasmic domain was expressed. This result reveals that TRAF3 degradation is not sufficient to induce BAFFR-mediated NF-κB2 activation. Results presented in Figures 6B and 7A led us to predict that TRAF recruitment to the A/WySnJ BAFFR cytoplasmic tail would be comparable to TRAF recruitment to Wt BAFFR. Indeed, cells expressing hCD40-A/WySnJ chimeric molecules not only were able to recruit both TRAF2 and 3 to the BAFFR, this mutant BAFFR showed enhanced TRAF recruitment compared to Wt BAFFR (Figure 7B). Consistent with previous studies, when the TRAF3 binding site (PVPAT) on BAFFR was disrupted (AVAAA), there was no recruitment of TRAFs to the receptor’s cytoplasmic domain (Figure 7B). These results show that the defective BAFF-stimulated NF-κB2 activation in A/WySnJ B cells is not due to a failure to induce TRAF3 recruitment or degradation.

Taken together, results from experiments presented in this study indicate that receptor-mediated TRAF3 degradation is not sufficient to induce NF-κB2 activation in B cells and TRAF3 degradation-independent mechanisms can also regulate the NF-κB2 pathway.

## Discussion

Functions of TRAF molecules vary both between cell types, and between individual types of receptors even within the same cell. This is particularly true for TRAF3 (2). There are many disadvantages of using the experimental model of transient overexpression in transformed cell lines, usually epithelial or fibroblast, to understand how endogenous signaling pathways work in specific hematopoietic cell types [reviewed in Ref. (47)]. Unfortunately, a great deal of the experimental investigation of TRAF3 functions to date has relied upon overexpression models. Largely based upon such models, it was concluded that receptor-induced TRAF3 degradation leading to NIK stabilization is a crucial requirement for downstream activation of the non-canonical NF-κB2 pathway (20, 26, 27).

We wished to further explore this model in B lymphocytes, as our previous findings, and those of others, indicate that TRAF3 plays uniquely important roles in regulation of this cell type. Our results presented here using complementary experimental systems of primary B cells and B cell lines, demonstrate that TRAF3 degradation is not sufficient for downstream NF-κB2 activation. The results from our study further emphasize the importance of studying TRAF functions in a cell type and receptor-specific manner. In this study, we also demonstrated TRAF-N and TRAF-C domains are required for recruitment of TRAF3 to CD40 and BAFFR, and that association with the TRAF2 complex is necessary for receptor-mediated TRAF3 degradation (Figures 1 and 4). However, the lack of degradation of the MM-derived TRAF3 mutant LP1 did not prevent activation of downstream signaling pathways; in fact, LP1-expressing B cells showed similar ability to undergo receptor-induced NF-κB2 activation, and enhanced JNK activation. In contrast to a previous study performed in non-hematopoietic cells (20), we found that TRAF3 association with the NF-κB2-activating kinase NIK in B cells does not require the TRAF-N and TRAF-C domains. Although lacking these domains, the LP1 mutant TRAF3 molecule actually bound substantially more NIK than Wt TRAF3. While the explanation for this enhanced binding is currently unknown, it is possible that one or more additional protein–protein interactions are involved. A slight increase in total NIK protein in LP1-expressing cells also suggested that NIK stabilization can be controlled by additional mechanisms. IKKα phosphorylation of NIK also destabilizes NIK (48). Additionally, TBK1 stabilizes NIK protein levels independent of TRAF3 degradation, while in contrast the protein OTUD7B regulates NIK protein levels through TRAF3 degradation in B cells (49, 50). Unlike TRAF3-deficient B cells, LP1 TRAF3-expressing B cells responded to both BAFF and CD40 stimulation with further increases in nuclear p52 levels. The current proposed model is that TRAF3 association with the TRAF2-cIAP complex prevents activation of the NF-κB2 pathway. Results presented here indicate that the mechanism by which TRAF3 regulates NF-κB2 activation is more complex than this. The degradation-resistant LP1 mutant TRAF3, without complexing with TRAF2-cIAP, can still regulate NF-κB2 activation. Our results also showed that the amino terminal RING and Zinc finger domains of TRAF3 are also quite important for TRAF3 to restrain NF-κB2 activation, in accordance with a published study (8). Taken together, the published and present data demonstrate that there are multiple mechanisms by which TRAF3 regulates the NF-κB2 pathway in B cells, both dependent upon, and independent of receptor-mediated TRAF3 degradation.

B cell activating factor receptor-mediated activation of signaling pathways is important for B cell homeostasis and survival, as exemplified by the deficiencies in mature B cell numbers and function observed in BAFF^−/−^ and BAFFR^−/−^ mice, as well as the spontaneous BAFFR mutant-expressing mouse, A/WySnJ (5). The NF-κB2 pathway is robustly activated by BAFFR, and is thought to play a major role in BAFFR-mediated B cell survival signals (21). Thus, the previously presented paradigm of NF-κB2 activation predicts that loss-of-function mutants of BAFFR would show reduced ability to induce TRAF3 degradation in B cells. Consistent with previous reports (28, 51), we observed impaired BAFF-mediated NF-κB2 activation in B cells isolated from A/WySnJ and BAFFR^−/−^ mice, as well as with B cell lines expressing mutant hCD40-BAFFR chimeric receptors. We further demonstrated that the last eight amino acids of the BAFFR cytoplasmic domain are required for NF-κB2 activation in B cells. This is particularly interesting, because Mayne and Hayes reported that these residues are NOT required for BAFFR-mediated B cell survival (29). Together with the present results, this indicates that NF-κB2 activation is not the only important pro-survival pathway induced by BAFFR signaling.

Several studies have concluded that receptor-induced TRAF3 degradation is sufficient to activate the NF-κB2 pathway (20, 26, 27). Because the A/WySnJ BAFFR retains the TRAF3 binding motif PVPAT, we compared BAFFR-inducedTRAF3 degradation using B cells expressing different forms of BAFFR. Again in contrast to the current paradigm, we found that signaling through the A/WySnJ or ΔC BAFFRs induced TRAF3 degradation to levels similar to Wt BAFFR, leading to the conclusion that receptor-induced TRAF3 degradation is not sufficient to drive NF-κB2 activation. Additionally, the A/WySnJ BAFFR recruits more TRAF2 and 3 to its cytoplasmic tail, so it is possible that the mutant BAFFR is recruiting important additional, unidentified signaling molecules that regulate NF-κB2 activation. We also found that the last eight amino acids of the C terminus of BAFFR were not required for either receptor-mediated TRAF3 degradation or TRAF recruitment to the receptor, but the impact of this region upon recruitment of other unidentified factors are not yet known.

In summary, our data demonstrate that the relationship between TRAF3 degradation and activation of NF-κB2 in B cells is neither simple nor linear. These findings support the need for further investigation into both how NF-κB2 activation is regulated in B cells, as well as how TRAF3 may regulate B cell survival via mechanisms additional to NF-κB2 activation. The identification of loss-of-function TRAF3 mutations in various types of human B cell malignancies underlines the importance of reaching a clearer and more detailed understanding of these relationships.

## Author Contributions

Wai W. Lin, Joanne M. Hildebrand, and Gail A. Bishop designed research; Wai W. Lin and Joanne M. Hildebrand performed experiments. Wai W. Lin, Joanne M. Hildebrand, and Gail A. Bishop prepared the manuscript.

## Conflict of Interest Statement

The authors declare that the research was conducted in the absence of any commercial or financial relationships that could be construed as a potential conflict of interest. The editor declared a potential conflict of interest to the main Topic Editor of this Research Topic, Dr. Linda Burkly, prior to handling this paper because the author Joanne Hildebrand and the editor John Silke are currently employed by the Walter and Eliza Hall Institute of Medical Research. Dr. Burkly was satisfied that the potential for conflict was minor. The editor, John Silke, now further declares that there has been no conflict of interest during the review and handling of this manuscript.
